# Digital Solutions to Diagnose and Manage Postbariatric Hypoglycemia

**DOI:** 10.3389/fnut.2022.855223

**Published:** 2022-04-07

**Authors:** Katja A. Schönenberger, Luca Cossu, Francesco Prendin, Giacomo Cappon, Jing Wu, Klaus L. Fuchs, Simon Mayer, David Herzig, Andrea Facchinetti, Lia Bally

**Affiliations:** ^1^Department of Diabetes, Endocrinology, Nutritional Medicine and Metabolism, Inselspital, Bern University Hospital, University of Bern, Bern, Switzerland; ^2^Division of Clinical Pharmacy and Epidemiology, Department of Pharmaceutical Sciences, University of Basel, Basel, Switzerland; ^3^Department of Information Engineering, University of Padova, Padova, Italy; ^4^Institute of Computer Science, University of St. Gallen, St. Gallen, Switzerland; ^5^ETH AI Center, Swiss Federal Institute of Technology (ETH) Zurich, Zurich, Switzerland; ^6^Technology Studies, School of Humanities and Social Sciences, University of St. Gallen, St. Gallen, Switzerland

**Keywords:** bariatric surgery, postbariatric hypoglycemia, postprandial hypoglycemia, Roux-en-Y gastric bypass, dumping syndromes, diet records, mobile applications, decision support systems

## Abstract

Postbariatric hypoglycemia (PBH) is an increasingly recognized late metabolic complication of bariatric surgery, characterized by low blood glucose levels 1–3 h after a meal, particularly if the meal contains rapid-acting carbohydrates. PBH can often be effectively managed through appropriate nutritional measures, which remain the cornerstone treatment today. However, their implementation in daily life continues to challenge both patients and health care providers. Emerging digital technologies may allow for more informed and improved decision-making through better access to relevant data to manage glucose levels in PBH. Examples include applications for automated food analysis from meal images, digital receipts of purchased food items or integrated platforms allowing the connection of continuously measured glucose with food and other health-related data. The resulting multi-dimensional data can be processed with artificial intelligence systems to develop prediction algorithms and decision support systems with the aim of improving glucose control, safety, and quality of life of PBH patients. Digital innovations, however, face trade-offs between user burden vs. amount and quality of data. Further challenges to their development are regulatory non-compliance regarding data ownership of the platforms acquiring the required data, as well as user privacy concerns and compliance with regulatory requirements. Through navigating these trade-offs, digital solutions could significantly contribute to improving the management of PBH.

## Introduction

Postprandial hypoglycemia after bariatric surgery, also referred to as postbariatric hypoglycemia (PBH), is an increasingly recognized complication of bariatric surgery. The condition manifests with hypoglycemic episodes 1–3 h after meals, particularly if containing fast-acting carbohydrates (1). Blood glucose management for PBH patients consists primarily of nutritional strategies. Emerging technologies support and assist patients in their nutritional management of PBH. The aim of this review is to give an overview of the role of continuous glucose monitoring (CGM) automated macronutrient estimation of meals, automated dietary intake estimations, and digital platforms for multi-level data integration and decision support systems for PBH.

## Prevalence, Pathophysiology and Clinical Manifestation of Postbariatric Hypoglycemia

Differences in diagnostic criteria yield varying prevalence estimates and recent results suggest that PBH affects approximately 30% of postbariatric patients (2), more frequently those who underwent Roux-en-Y gastric bypass (RYGB) surgery (3). Additionally, many patients with PBH are asymptomatic which suggests that prevalence among the postbariatric population may be even higher (2, 4–6). If present, symptoms in PBH patients include autonomic (e.g., trembling, anxiety, palpitations, sweating) and neuroglycopenic symptoms (e.g., fatigue, concentration difficulties, confusion, vision changes). Severe hypoglycemia can lead to seizures, loss of consciousness, falls, motor vehicle accidents, and even death. Associated disability and compromised quality of life can be profound, and the condition does not appear to remit over time. Due to the increasing use of bariatric surgery for durable resolution of obesity and diabetes (7), clinicians should be familiar with the condition, including its clinical management, and improved methods to manage PBH are required.

Defining a clinically important glucose threshold is critical for diagnosis, quantification of disease severity and indication for intervention. A glycemic threshold of <3.0 mmol/L is deemed to be clinically meaningful as it is associated with the occurrence of neuroglycopenic symptoms and sequelae in patients with diabetes (8). It was recently shown that this threshold also applies to the PBH population (9). Finally, in the light of the high incidence of hypoglycemia unawareness and the low sensitivity of symptoms to hypoglycemia, it was recently suggested that the presence of neuroglycopenic symptoms may disappear over time and should therefore not be a requirement to diagnose PBH (5).

**Figure 1:**
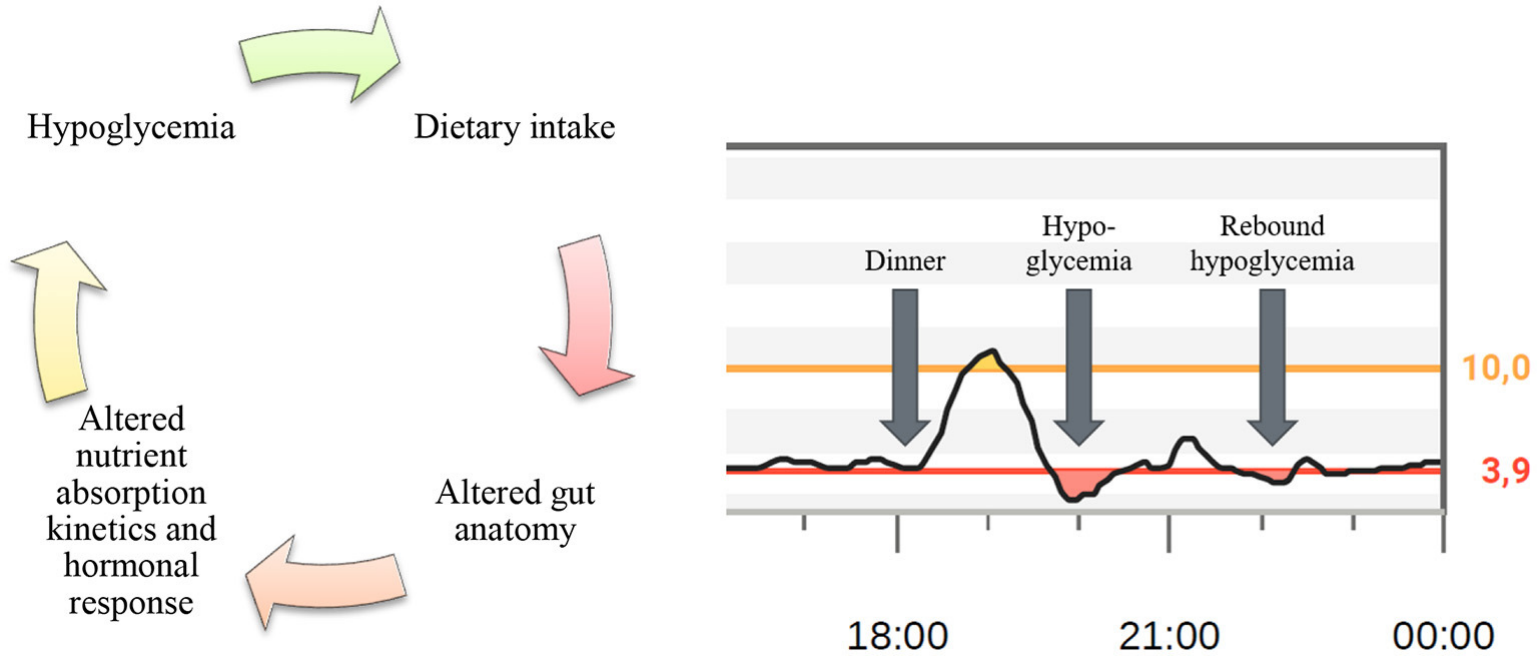
Origin of postbariatric hypoglycemia. Interaction between dietary intake and hypoglycemic events in patients with postbariatric hypoglycemia (left) and a section from a CGM profile showing postprandial hypoglycemia followed by rebound hypoglycemia caused by the correction of the primary hypoglycemia (right).

Although the pathophysiology of PBH is only partially understood, the excess postprandial insulin exposure in PBH patients is likely driven by accelerated nutrient absorption kinetics and stimulation of insulinotropic gut factors such as glucagon-like peptide-1 (GLP-1) because of the altered gastrointestinal anatomy (10). The rapid delivery of nutrients into the jejunum, particularly after RYGB, causes a prompt appearance of glucose in the blood. It has been shown that after RYGB, during the first hour following the ingestion of 75 g of glucose, approximately 45% of the total ingested amount appears in the circulation compared to about 30% pre-surgery (11). Entero-plasticity with the adaptation of intestinal epithelium and change in glucose transporter density can further accelerate systemic glucose appearance over time (12). The resulting early postprandial glucose peak exerts a stimulatory effect on the pancreatic cell, which is further amplified by insulinotropic gut factors, a phenomenon known as the incretin effect (1, 13–15). This observation is further supported by evidence demonstrating that administering the same amount of carbohydrate via a gastrostomy in the bypassed stomach leads to a total remission of PBH symptoms and normalization of pathologic glucose and insulin curves as the same meal given via oral intake (16). Other mechanisms, such as diminished neuroendocrine counterregulation, reduced insulin clearance and altered bile acids may further contribute to PBH (17–19). Figure 1 summarizes the interaction between dietary intake and hypoglycemic events in PBH patients.

## Nutritional Management of Postbariatric Hypoglycemia

Since prompt absorption of dietary glucose is a root cause of PBH, it seems reasonable that diet modification represents the first-line therapy of PBH. This is further supported by the current absence of an approved pharmacotherapy to treat the condition. With the aim to diminish postprandial glycemic excursions, the key nutritional management concepts include restriction of the carbohydrate load, choice of low glycemic index carbohydrates and consistent combination with other macronutrients such as protein and fat. In terms of carbohydrate intake, it was shown that limiting a meal to 30 g of solid carbohydrate or 28 g of liquid low glycemic index supplement was successful in preventing hypoglycemia in patients with PBH (20). Another study recently demonstrated that a compensatory increase in protein content of a meal raises the nadir plasma glucose concentration by 13% and was accompanied by reductions of GLP-1, gastric inhibitory polypeptide (GIP), and insulin as well as increases in glucagon concentrations (21). Fats can also serve as a substitute calorie source to compensate for the reduction in carbohydrates. Fats do not typically trigger insulin secretion and may even induce some protective transient insulin resistance thereby stabilizing postprandial glycaemia (22). For PBH patients, recommended macronutrient distributions typically are 30% carbohydrates, 45–50% fats and 20–25% protein (higher protein if weight reduction is an additional goal). Further nutritional strategies focus on a high fiber intake (natural foods or dietary supplement such as glucomannan, guar or pectin) to reduce the absorption rate of dietary glucose and diminish postprandial glycemic excursions (23–25). Finally, fluid intake should generally be separated from the main meal and semi-solid or liquid dishes should be limited due to their more rapid absorption kinetics. Table 1 gives an overview of nutritional strategies for the management of PBH.

**Table 1:** Dietary modifications for the management of postbariatric hypoglycemia.

**Aspect**	**Dietary modification**
Carbohydrates: Quantity	- ≤30 g carbohydrates per meal - Several small meals spread throughout the day
Carbohydrates: Quality	- Starch sources high in fiber (whole grains, legumes) - Avoidance of rapidly absorbed carbohydrates (sugar/sweets) or replacement with sugar-free options
Protein and fats	- Consistent combination of carbohydrates with foods high in fat and protein and vegetables/salad - Protein/fat-rich foods should ideally provide >70% of meal energy - Specific amino acids to increase endogenous glucagon (e.g., arginine)
Meal pattern	- Dessert/snack 90 min after meals (to offset rapid blood glucose falls) - Avoidance of liquids together with meals
Soluble dietary fibers	- Addition of soluble dietary fibers to slow down carbohydrate absorption (guar/pectin)

Another component of nutritional management is to improve safety in patients with PBH by adequate correction of hypoglycemic events. Previous research suggests that glucose co-ingested with amino acids induces a metabolic environment that could be favorable for PBH patients due to elevated glucagon levels (21, 26). However, it currently remains speculative whether combinations of amino acids with glucose could offer more suitable and sustainable hypoglycemia correction strategies. Another strategy, although speculative in the PBH population today, is intake of caffeine (3–6 mg/kg), which is known to stabilize glycaemia via induction of peripheral insulin resistance and possibly increase of endogenous glucose production (27, 28). It is important to note that standard dietary advice given to individuals with diabetes whose gastrointestinal tract is intact is not applicable to the circumstances in PBH patients. This is illustrated by current diabetes-inspired guidelines recommending hypoglycemia correction with 15–20 g of fast-acting carbohydrates, preferably glucose (29, 30). However, clinical experience with PBH patients shows that the rapid spikes in glycaemia following correction of hypoglycemia with such strategies can trigger rebound hypoglycemia. Guidelines on hypoglycemia correction strategies that are tailored to the specific needs of PBH patients do not exist to date.

## The Role of Continuous Glucose Monitoring

PBH patients have rapid meal-induced fluctuations in glucose concentration levels in both directions with early postprandial blood glucose spikes in the diabetic range followed by a sharp decrease leading to hypoglycemia in the late postprandial period. With the advent of CGM, which allows for measuring interstitial glucose levels every 5 min, real-time visualization of glycemic trajectories has become possible. CGM devices transmit glucose measurements at regular intervals from a wearable body sensor to a nearby receiver or mobile device through a low-power wireless technology (e.g., Bluetooth Low Energy), providing users with actionable information on historic and current glucose concentration and velocity of glucose change. Whilst the technology has become the standard glucose monitoring tool for patients with diabetes (31), its use in the PBH population is currently still considered off-label. However, CGM sensors may provide remarkable benefits for the management of PBH in clinical practice. Not only can the immediate and precise capturing of individual meal-induced glycemic fluctuations improve dietary choices and behavior through a trial-and-error approach, but CGM is furthermore useful for detecting asymptomatic but clinically significant hypoglycemic events (glucose <3.0 mmol/L according to the International Hypoglycaemia Study Group). In a recently published study, for example, CGM captured up to 10-fold more events than were captured by symptom-driven capillary blood glucose measurements (9). Customizable alerts can prompt patients for preventive and corrective actions and allow them to review such actions critically (e.g., prevention of overcorrection resulting in rebound hypoglycemia).

CGM data can also be used to develop algorithms to predict future hypoglycemia in real-time. Prediction methodologies employed so far for such a purpose include classical time series-based forecasting methods as well as machine learning and deep learning techniques (32–34). Incorporation of contextual data (e.g., meal information, physical activity) as features in the predictive model are also being explored (35–37). Predictive hypoglycemia alerts have the potential to substantially reduce the burden of hypoglycemia, however, false alarms can be a major hindrance of their acceptance among users (38). While most of the work on CGM-based hypoglycemia prediction has been done in the diabetic population, research in PBH patients is currently scarce (39).

Despite its potential, the current state of CGM technology is not without limitations. Although the latest generation CGM devices provide satisfying accuracy at steady state conditions, CGM glucose levels may lag behind blood glucose concentrations when glucose is changing rapidly, as in the postprandial state (40). Additionally, the accuracy of CGM in the hypoglycemic range is currently not satisfying and patients are advised to confirm low levels with capillary blood glucose measurements (41). Finally, current devices are not designed to handle the rapid glucose dynamics that are characteristic for PBH patients and cause gaps in data visualization and default non-mutable low thresholds are set above the clinically relevant threshold for PBH thereby risking nuisance for patients. A further drawback is that patients need to be provided with CGM instrumentation that they need to wear in addition to other sensors that they already have. A preferable approach would be to find CGM-like signals from data that the patients already collect with the devices that they already have (e.g., heart rate and accelometer data from smartphones and smartwatches).

## Digital Solutions To Support Dietary Decision Making

Existing evidence and clinical experience highlight the potential for nutritional strategies to manage PBH in daily life and overcome potentially debilitating consequences (21, 42). However, practical implementation of such nutritional strategies is not without challenges. First, substantial nutritional knowledge, including carbohydrate counting skills and literacy regarding carbohydrate quality (e.g., glycemic index) is necessary to follow current guidelines. In addition to the required knowledge it is also simply time that the patients need to invest. The considerable manual effort that is involved can lead to high churn rates. Second, it is important to recognize that there is substantial variability in glucose profiles between individuals, and even from day to day within the same patient, potentially related to rate of delivery of foods to the intestine, rates of glucose absorption by the proximal intestine, time of day, and other metabolic factors (e.g., physical activity). As a consequence, nutritional goals and measures to achieve these goals need to be continuously adapted upon review of dietary and glucose patterns.

Digital technologies offer the potential to overcome these challenges and facilitate dietary management of PBH patients whilst reducing the burden of hypoglycemia. Mobile applications and wearable technologies (e.g., CGM sensors and smart watches) provide opportunities for real-time collection of granular health and nutrition-related data. Artificial intelligence, machine and deep learning methodologies can be employed to process and analyze the increasing amount of collected data. The combination of these approaches can be translated into practical clinical applications, such as decision support, risk prediction and diet optimization tools. Whilst many diet applications are existing, very few are suitable to meet the specific and complex needs of PBH patients. In the following section, we propose a selection of novel digital solutions that may support the nutritional management of PBH patients in daily life.

### Automated Macronutrient Estimation

**Figure 2:**
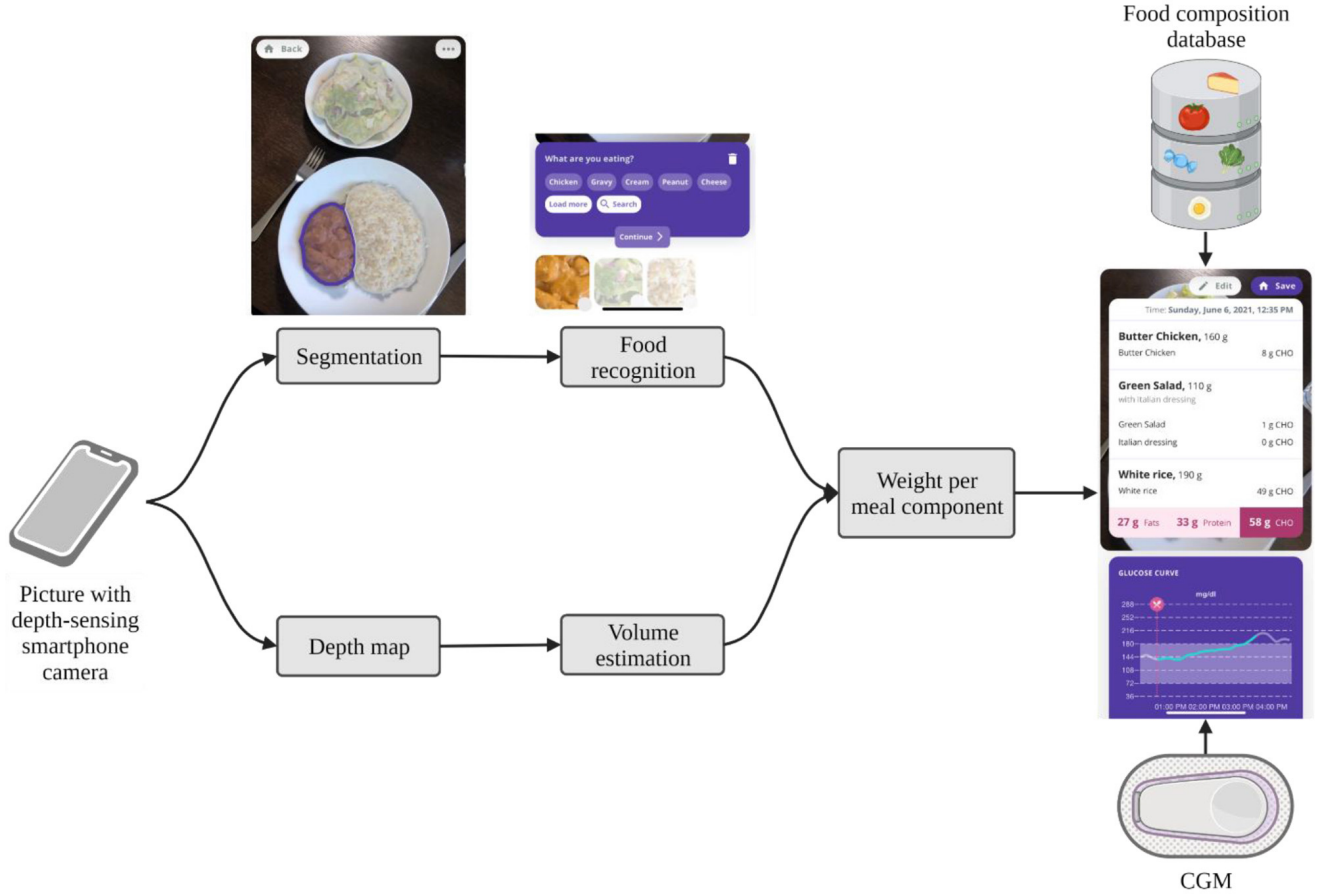
Technical flow of the SNAQ app. The app automatically segments pictures of meals into meal components and recognizes the food of the components. If the picture is taken with a depth-sensing camera of newer smartphones, it creates a depth map to estimate the volume of each meal component. From the volume and the food recognition, it can calculate the weight of each meal component. By using a food composition database, the macronutrient composition of the meal is calculated and displayed to the user with the corresponding CGM data. Screenshots kindly provided by SNAQ.

The quantification of macronutrients, particularly carbohydrates, is an important component of nutritional management in PBH. However, carbohydrate counting is a challenging task and prone to errors, even for trained patients with type 1 diabetes (43–46). With the development of computer vision algorithms in combination with pervasive smartphone cameras, meal macronutrient estimation from analyzing images captured from smartphone cameras has become feasible. Automated macronutrient estimation is usually generated by a three-stage process: (1) food item segmentation; (2) food item recognition and (3) volume estimation. Once the food item is recognized, nutrient content can be retrieved from a food nutrient database which contains both nutrient and density information. In line with this concept, many algorithms have been proposed in the scientific literature and some of these are accessible in the form of mobile applications. However, the great majority of existing algorithms focus on segmentation and recognition only, without providing information on quantity, which represents the main difficulty on the end users' side. This gap is explained by the technical challenge of estimating food volume. Nevertheless, in recent years multi-view geometry-based solutions have been developed requiring the patients to follow a specific photoshoot protocol to work. GoCARB uses the Canny edge detector and incremental random sample consensus (RANSAC) paradigm for plate detection and then hierarchical k-means that are fed into a support vector machine (SVM) for food classification (47). GoFood replaced GoCARB's SMV with a neural network (48). Today, also single-view methods (e.g., combined with depth data) are available (49). In this case, only a single image instead of a video or reference map is required. An example of the technical workflow of such a single-view method is displayed in Figure 2. Systems that additionally provide automated quantification of macronutrients were shown to provide accurate macronutrient estimations with absolute errors of 14% for weight and 15% for carbohydrate content (49). It is of note that the reported estimate is better than currently reported carbohydrate counting skills in the type 1 diabetes population (50) and was recently demonstrated to be comparable to dieticians' estimates (48, 51). However, accuracy results from controlled laboratory environments may overestimate performance in real word scenarios characterized by innumerable types of food items and shapes. Further challenges to the technology include mixed dishes (e.g., stews), drinks and semi-solid meals (e.g., yogurt/creams) as well as lacking availability of food density information in nutrient databases and macronutrient variability within food categories (depending on the preparation).

**Figure 3:**
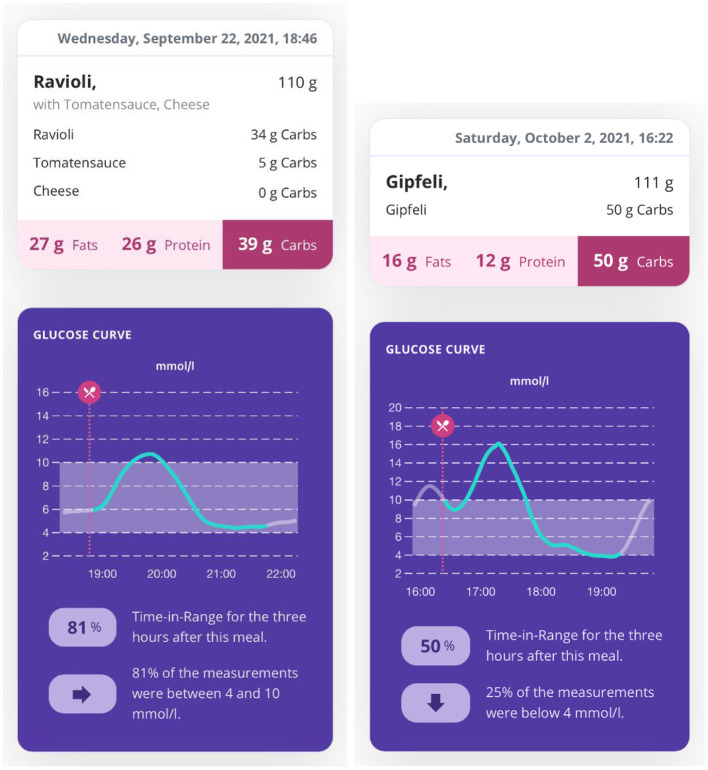
Output of image-based automated food assessment and corresponding glucose profiles. The SNAQ app allows for automated analysis of meal macronutrients from photographed meals. Pairing with a continuous glucose monitor combines the meal information with the corresponding postprandial glucose profile.

In a first technology concept testing, 8 patients with confirmed PBH used the SNAQ app within the framework of usual care nutritional counseling. Figure 3 illustrates two different meal assessment scenarios [(I) dinner consisting of ravioli with tomato sauce and cheese and (II) a croissant] with the corresponding glucose curve from a linked CGM. First experiences from both healthcare provider and patient perspectives suggest that the technology may be promising as it obviates the need for time-consuming and error-prone food diaries, provides real-time information of dietary intake and allows for personalizing dietary strategies when linked with CGM (e.g., assessment of individual carbohydrate thresholds in specific meal constellations). More comprehensive usability testing and clinical efficacy trials are required to fully evaluate its potential for the management of PBH.

### Digital Receipts for Grocery Choices With Optimized Glycemic Impact

Dietary intake and nutritional habits are greatly influenced by choices made at the grocery store. The great diversity of food products, the high turnover and often misleading front package claims (52) complicate the choice of products that fit the dietary recommendations for PBH. Even with existing food knowledge, checking food labels of different products and comparing these to each other is tremendously time-consuming (53). Thus, while basic feasibility of nudging shoppers toward healthier choices in physical (54) as well as online (55) stores has been demonstrated, optimizing food choices in real-life still requires technological innovations.

Digital receipts from loyalty cards have a high potential as an automatic, self-updating, and scalable approach to provide automated insights into the nutrient profile of food products and development of personalized recommendations, especially in countries where loyalty card programs are widely used (56–58). In Switzerland, for example, the loyalty programs offered by Coop and Migros, the two main grocery stores, reach more than 3 million households each, and 80% of the retail revenues are associated to loyalty cards (59, 60). Digital receipts provide shopping data that only includes product names, prices, and date of purchase. Hence, sales data first needs to be enriched with nutritional facts, which requires the creation, maintenance and adaptation of a product ingredient database (61). For example, the Auto-ID Labs of ETH Zurich and University of St. Gallen have developed and maintained a product ingredient database, which now contains more than 50,000 of the most frequently bought products in Switzerland (61). Its content reflects information that is contained in products' food facts and labels, e.g., energy, total fat, saturated fat, cholesterol, total carbohydrates, fibers, sugars and sodium as well as additional specifications such as a list of ingredients and allergens. Such food databases can be further expanded by self-designed metrics that are not visible on food products, e.g., a systematic methodology to estimate added sugar values on the basis of analytical data and ingredients of foods (62). Additionally, calculation of carbohydrates to fiber ratio allows to stratify carbohydrates according to their glycemic impact. Carbohydrate to fiber ratios of 10:1 are linked to foods with higher dietary fiber and lower free sugars (63).

Digital receipts from food purchases can be used for nutritional management in two different ways. They enable automated, continuous, objective and non-intrusive monitoring of purchased food items including analysis of the distribution into specific food categories and breakdowns into macronutrients and other nutritional properties. Of note, more holistic metrics that provide insights into the overall healthiness and nutritional quality of the purchased food have been developed. Examples include the Food Standards Agency Nutrient Profiling System Dietary Index (FSA-NPS DI) (64), the Healthy Trolley Index (65) or the Grocery Purchase Quality Index-2016 (66). A comparison between these food shopping quality indicators found that correlations between food shopping data and density-based relative food and nutrient intake are stronger than absolute food and nutrient intake (58). Unlike self-reported dietary intake, which assesses individual food intake, food items recorded with digital receipts are typically consumed by a whole household. However, a reasonably accurate conversion of household-level food purchase data to individual-level data can be achieved by using expenditure (66, 67) or caloric shares (68). Limitations of this method include differences between what is bought and what is eaten, non-tracked consumption, and distribution among family members.

**Figure 4:**
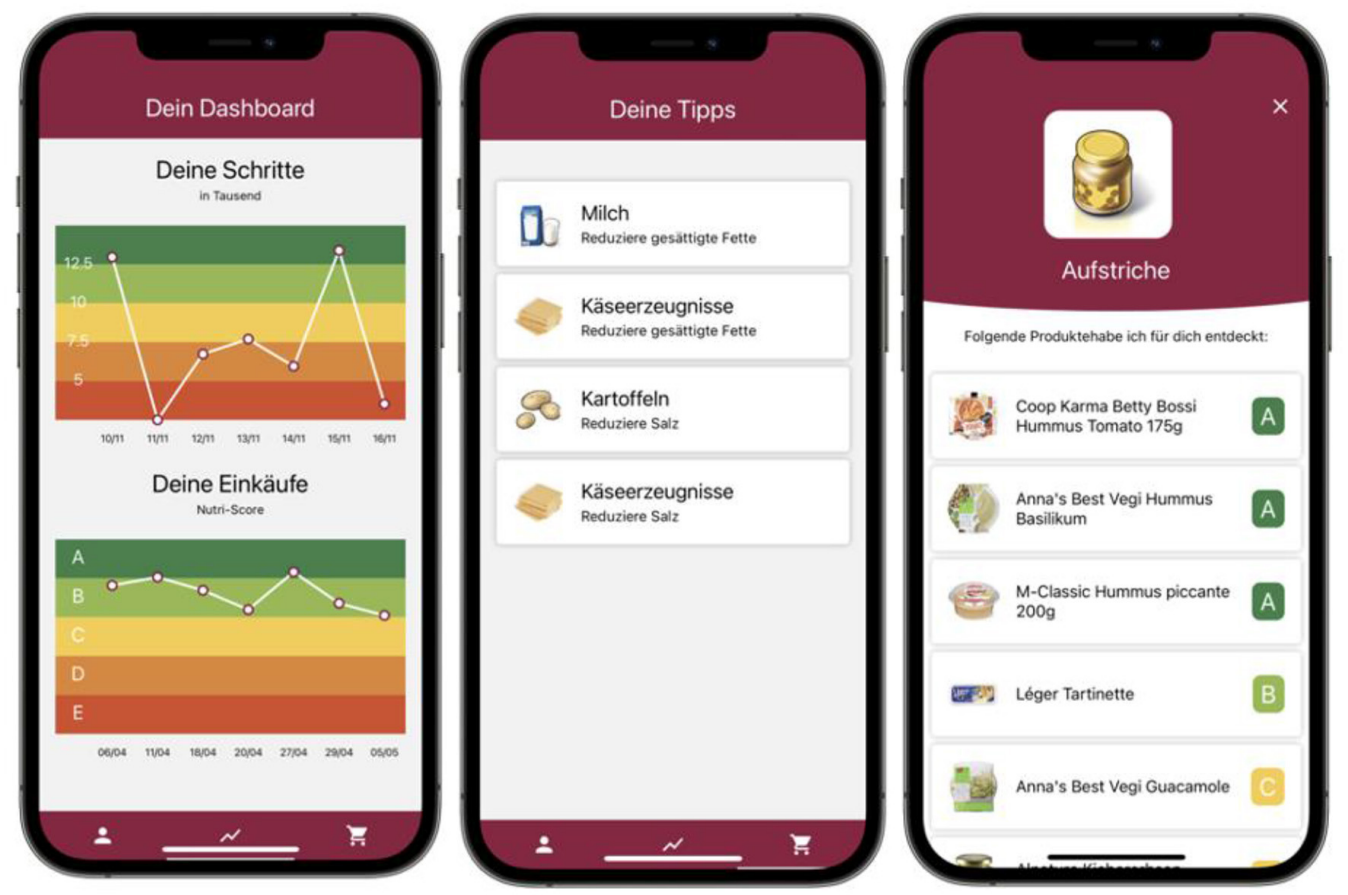
Mobile application using digital receipts to optimize diet. The app screenshots provide insights into the functionalities of the app: daily overall nutritional value of purchased food (e.g., using the Nutri-Score) (left); food categories for individualized goal setting (center); food recommendations based on individual food purchase history and goal setting. The app currently exists only in German.

Apart from permitting the automated analysis of purchased food, digital receipts and linked food composition databases allow for setting nutritional goals and translation into personalized food recommendations delivered by dedicated mobile applications. For example, a nutritional goal for a PBH patient might be to consume food products that provide a maximum of 30 g carbohydrates per serving size, or cereals with a certain carbohydrate-to-fiber ratio. One example of a digital receipts-based product advice application is FutureMe (69). Figure 4 shows an example of using digital receipts to assess people's shopping behavior and provide detailed recommendations.

Despite its promises, the most relevant barriers in the adoption of digital receipts are individual user burden, retailer resistance, lack of standardization, infrastructure and data privacy. Not all retailers have a loyalty card program and loyalty cards do not record the whole consumption, such as when customers forget the card, food waste, or eating out. In addition, customers may share their loyalty cards with other people. Challenges regarding the infrastructure include maintenance of the food composition database (adding new products), linking digital receipts with nutritional information, and missing unique identifiers on receipts (only prices and names). This could be solved through standardization of the receipts to make items identifiable, such as with the global trade item number. Finally, people may be reluctant to share their data. However, it can be expected that retailers will mass-adopt the distribution of digital receipts, once they are required by regulation or when they become a de-facto standard for cashless payments.

### Digital Platforms for Multi-Level Data Integration and Decision Support

Nutritional management in PBH lends itself to interactions centered around data—information on meals, blood glucose values and trajectories, symptoms, physical activity and possibly off-label pharmacotherapies. Digitalization can enhance PBH care not only through the improved collection and analysis of data, but also through their connection in the form of integrated platforms. This allows both patients and healthcare providers to evaluate the collected data, both in real time and retrospectively. The ability to simultaneously view both retrospective and real-time data from multiple sources allows evaluating cause-and-effect relationships among diet, glucose trajectories and other factors such as physical activity. Such analysis is a key component to optimize the clinical management of PBH as it sets the foundation for informed decision making. Apart from facilitating and optimizing clinical management, integrated platforms can support large-scale data collection (e.g., in clinical trials) which can be used for the development, training and validation of hypoglycemia prediction models. Finally, such predictive models can be incorporated into data-driven decision support systems, which can inform patients about imminent hypoglycemia and ultimately about optimized dietary choices to improve glucose control. Pioneering work by Zeevi et al. (70) has provided proof of principle for the utility of tailoring nutrition to individual glucose profile, albeit not in PBH patients.

**Figure 5:**
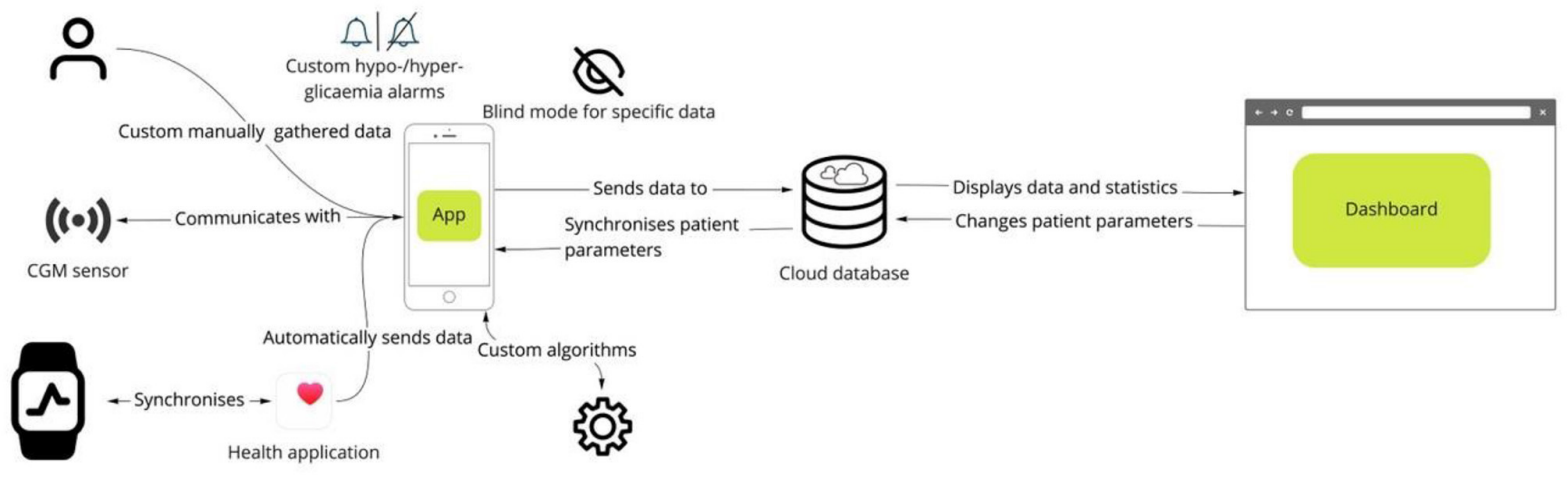
Digital platform for multi-level data integration. The platform gathers data from multiple sources, such as manual input, continuous glucose monitoring sensors and health data from smartwatches. Custom algorithms and personalized data visualization (e.g., blinding data for specific classes of patients) allow for tailoring the functionalities to individual needs. The dashboard allows the healthcare professional to monitor data acquisition in real time and optimize treatment.

**Figure 6:**
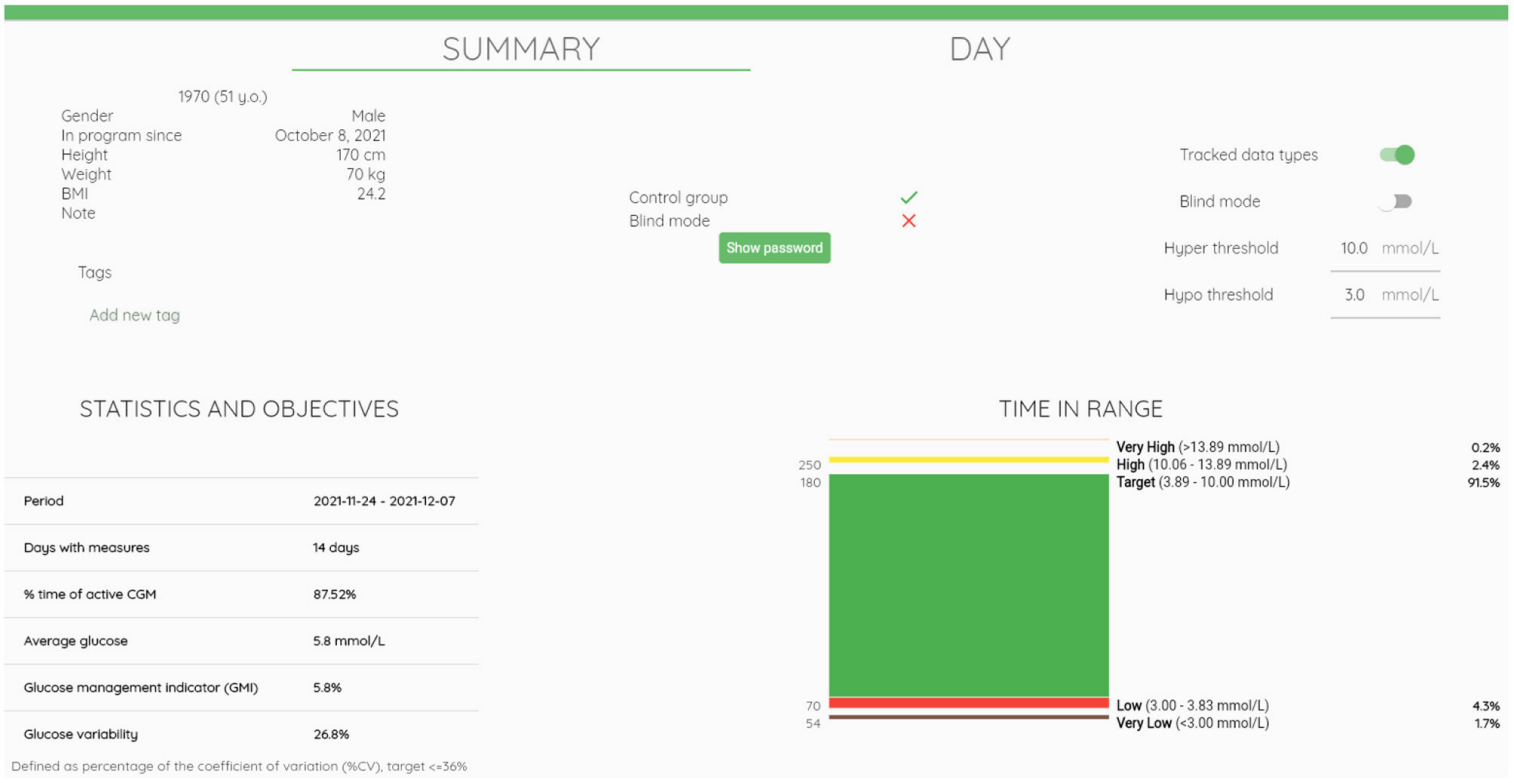
Browser dashboard summary view. The browser dashboard for the healthcare professional provides an overview of patient characteristics, settings for data collection, data view and notifications. It further displays summary statistics of continuous glucose monitoring data.

**Figure 7:**
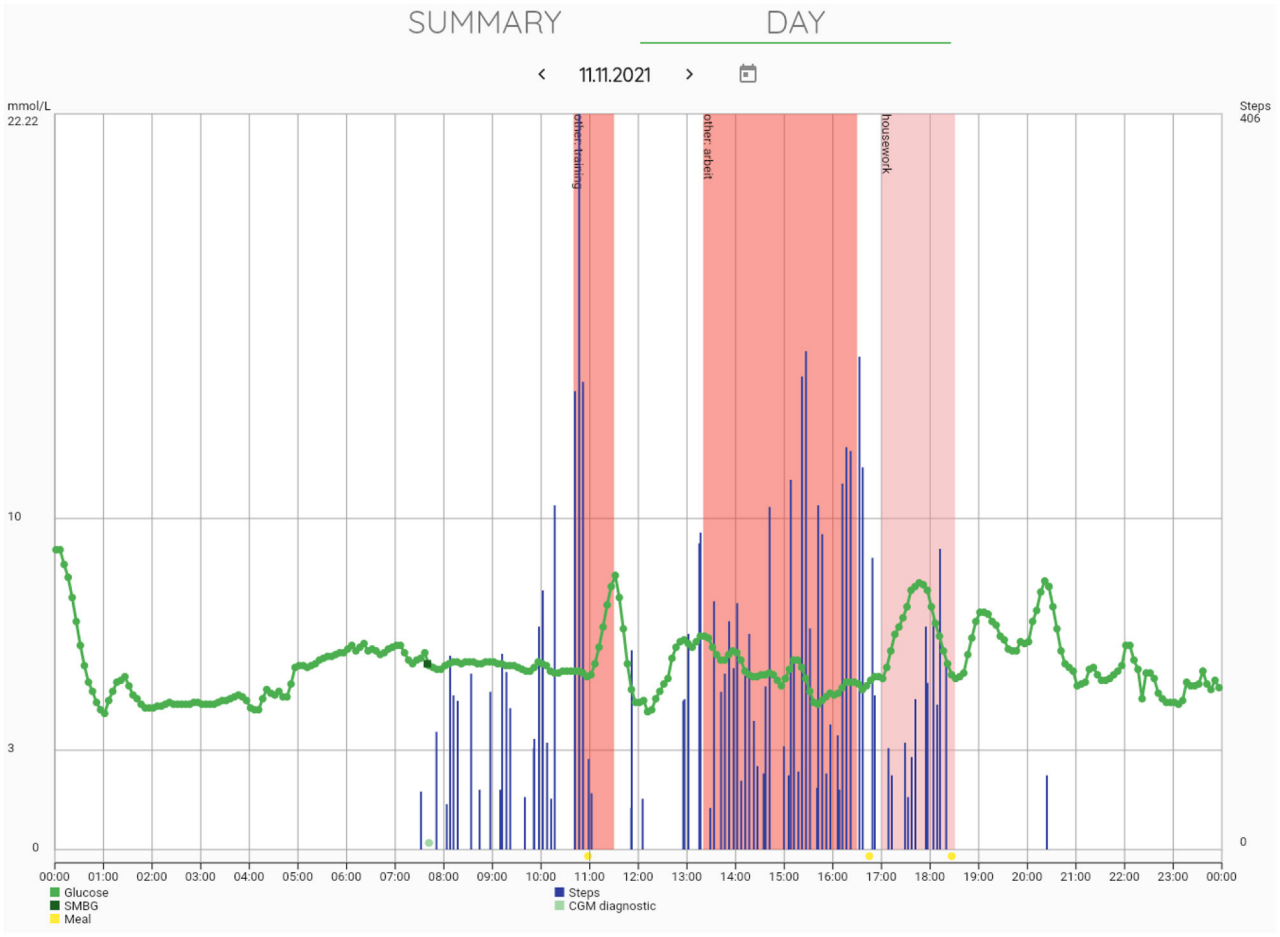
Browser dashboard daily view. The browser dashboard further allows for day-by-day review of glucose trajectories that are displayed in combination with other types of data collected such as self-measured blood glucose (SMBG), step counts from activity trackers, and physical activity logs and meal logs entered by the user.

**Figure 8:**
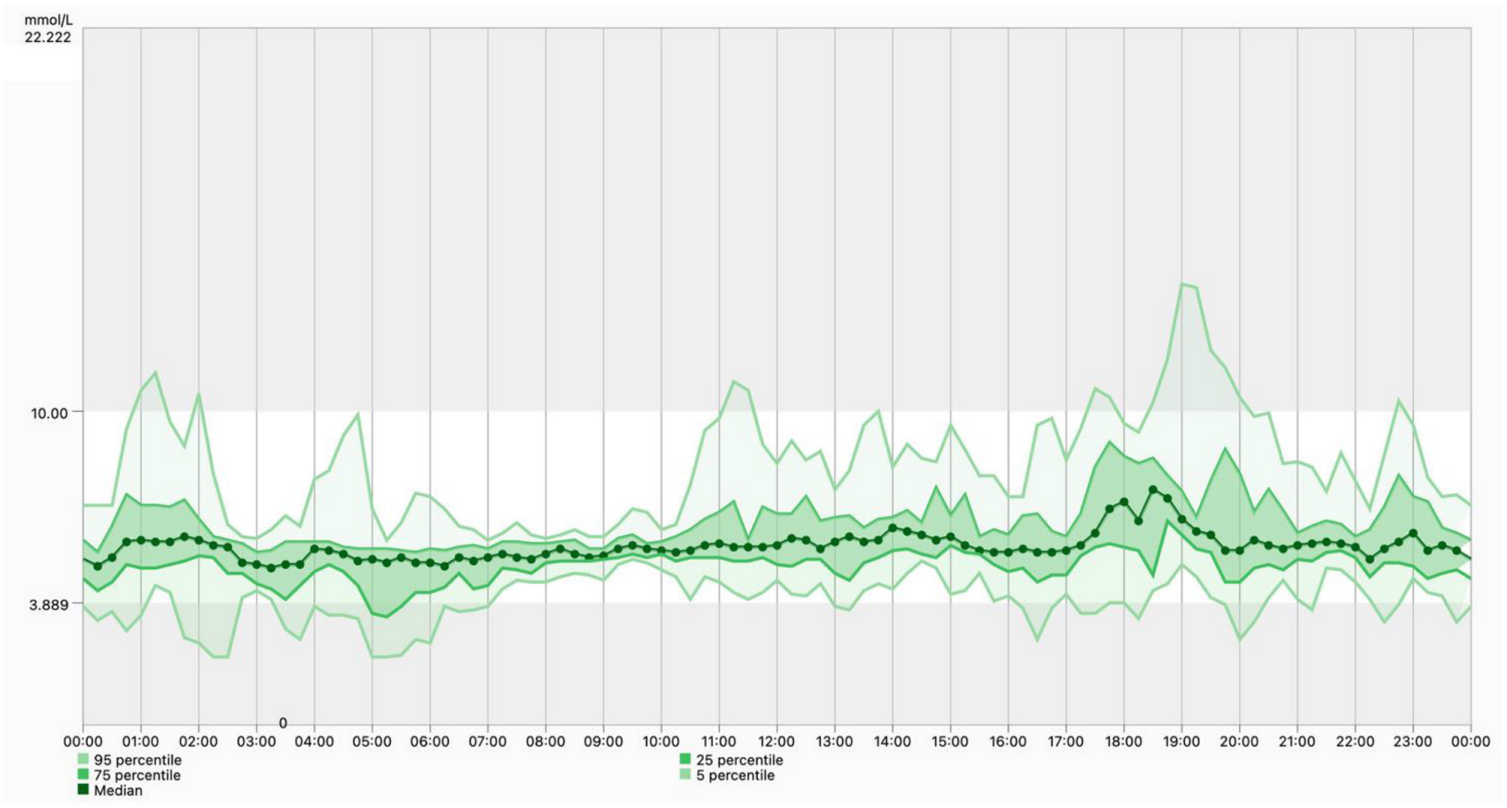
Ambulatory glucose profile. The dashboard includes a standardized visual report once the software has sufficient numbers of days of data collection. It shows a median glucose control line; the 25th−75th percentiles, which represents 50% of the glucose readings over the analysis time period; and the 5th−95th percentiles, which helps identifying outliers that are contributing to the median results.

An example of an integrated mobile platform, developed by the Department of Information Engineering of the University of Padova, is illustrated in Figure 5. The system has originally been created for patients with diabetes (71) and was modified, in collaboration with the Department of Diabetes, Endocrinology, Nutritional Medicine and Metabolism of the University Hospital Bern, to meet the specific needs for clinical research in PBH patients and for future use in clinical practice. The components of the platform, which consists of a mobile app for patients, a web interface for researchers and healthcare professionals, a cloud database, and wearables integration work in synergy to ensure a secure environment for data collection and real-time monitoring. The mobile app integrates manual data input (e.g., tracking of meals, symptoms, drugs and events), data from the CGM device as well as other wearables (e.g., activity tracker), whereas the web interface allows researchers and healthcare professionals to access data in real time on a browser dashboard (Figure 6). Figure 7 illustrates a daily glucose profile and Figure 8 a summary statistics over several days.

The main challenge in the development of such platforms is device interoperability, which refers to the ability of devices to connect and exchange data. Although improved over the past years, siloed systems continue to complicate the creation of connected ecosystems.

## Remaining Challenges of Digital Approaches

As wearable devices transition from lifestyle monitoring to medical devices (where data acquired from the devices are the basis for medical decisions), they will be subject to greater regulation (72). Therefore, the United States Food and Drug Administration (FDA) created the Digital Health Software Precertification (Pre-Cert) Program for low-risk device approval (73). This program will “provide more streamlined and efficient regulatory oversight of software based medical devices developed by manufacturers who have demonstrated a robust culture of quality and organizational excellence, and who are committed to monitoring real-world performance of their products once they reach the U.S. market.” A recent review found that the most important concerns with wearable devices are security and safety, particularly when data from wearable devices are used to make medical decisions (74). A major advantage of using wearable devices for health assessments is that the information is objective and the burden of self-reporting is eliminated. Clinical interventions with wearables provide users and clinicians with real-time information and can support behavior change. For consumer-based wearable devices to become more widely used in clinical practice, secure data sharing technology needs to be used properly and intermediaries need to be minimized. Ultimately, clinicians play an essential role interpreting data from wearable devices. It is thus important that clinicians are familiar with wearable devices and their limitations. In addition, the implementation of digital technologies for the management of PBH can lead to financial burden for patients and insurers. However, additional technology costs may be justified given the potential reduction of disease burden and improved patient safety.

## Conclusion

PBH is an increasingly recognized complication of bariatric surgery, for which nutritional management is the cornerstone treatment. Digital technologies, through the improved collection and analysis of data from multiple sources, hold great promise for supporting, optimizing and personalizing the management of diet and food in daily life whilst increasing quality of life and safety in affected patients.

Promising approaches include automated image-based food analysis, visualization of food effects using CGM, digital receipts for smarter food choices at the grocery store, integrated platforms to combine multiple sources of data and advanced data analysis techniques to develop better prediction algorithms and decision support systems.

However, several challenges remain that prevent digital technologies from reaching their full potential in the management of PBH and related conditions. From the users' perspective, the most important criteria for the uptake of such technologies is user burden, including effort for manual data input and device burden. Another key point in the development of digital solutions is how exactly a data controller complies with data security and privacy policies, such as the EU General Data Protection Regulation (GDPR). Additionally, it needs to be considered that depending on the functionalities and use of the algorithms, digital solutions may qualify as medical devices and are subject to regulation (e.g., Medical Device Regulation in Europe). International organizations, including the International Medical Device Regulators Forum and the World Health Organization (WHO), have made strides in classifying different types of digital health technology and integrating digital health technology into the field of medical devices. Although the stage of digital technologies to support nutritional management in patients with PBH is still young, there is much promise for growth, optimization and disruption in future. To integrate digital decision support systems into the everyday life of PHB patients, we need to ensure that they are based on the best evidence for safety, efficacy and utility. Such efforts require intensive collaborations between technical experts, clinicians, regulatory experts and device makers.

## Author Contributions

KS and LB conceptualized the work and wrote the first draft of the manuscript. LC and JW contributed to sections of the manuscript. LC, FP, GC, JW, KF, SM, and AF are involved in the development and maintenance of the described digital solutions. KS, DH, and LB provided input for user requirement specifications. All authors contributed to manuscript revision, read, and approved the submitted version.

## Funding

We acknowledge the support from the Swiss National Science Foundation (PCEGP3_186978), the Department of Diabetes, Endocrinology, Nutritional Medicine and Metabolism, Inselspital and a third-party grant of the Division of Clinical Pharmacy and Epidemiology, University of Basel, Grant Number FO119900. This work was supported by the Department of Information Engineering, University of Padova (Italy) under the initiative SID-Networking Project 2021 (DVTDSS project).

## Conflict of Interest

The authors declare that the research was conducted in the absence of any commercial or financial relationships that could be construed as a potential conflict of interest.

## Publisher's Note

All claims expressed in this article are solely those of the authors and do not necessarily represent those of their affiliated organizations, or those of the publisher, the editors and the reviewers. Any product that may be evaluated in this article, or claim that may be made by its manufacturer, is not guaranteed or endorsed by the publisher.
